# Massive annotation of bacterial l-asparaginases reveals their puzzling distribution and frequent gene transfer events

**DOI:** 10.1038/s41598-022-19689-1

**Published:** 2022-09-22

**Authors:** Andrzej Zielezinski, Joanna I. Loch, Wojciech M. Karlowski, Mariusz Jaskolski

**Affiliations:** 1 Department of Computational Biology, Faculty of Biology, A. Mickiewicz University, Poznan, Poland; 2 Department of Crystal Chemistry and Crystal Physics, Faculty of Chemistry, Jagiellonian University, Krakow, Poland; 3 Department of Crystallography, Faculty of Chemistry, A. Mickiewicz University, Poznan, Poland; 4 Institute of Bioorganic Chemistry, Polish Academy of Sciences, Poznan, Poland

**Keywords:** Molecular evolution, Bacterial genomics, Bacterial evolution, Protein structure predictions, Protein sequence analyses

## Abstract

l-Asparaginases, which convert l-asparagine to l-aspartate and ammonia, come in five types, AI-AV. Some bacterial type AII enzymes are a key element in the treatment of acute lymphoblastic leukemia in children, but new l-asparaginases with better therapeutic properties are urgently needed. Here, we search publicly available bacterial genomes to annotate l-asparaginase proteins belonging to the five known types. We characterize taxonomic, phylogenetic, and genomic patterns of l-asparaginase occurrences pointing to frequent horizontal gene transfer (HGT) events, also occurring multiple times in the same recipient species. We show that the reference AV gene, encoding a protein originally found and structurally studied in *Rhizobium etli*, was acquired via HGT from *Burkholderia*. We also describe the sequence variability of the five l-asparaginase types and map the conservation levels on the experimental or predicted structures of the reference enzymes, finding the most conserved residues in the protein core near the active site, and the most variable ones on the protein surface. Additionally, we highlight the most common sequence features of bacterial AII proteins that may aid in selecting therapeutic l-asparaginases. Finally, we point to taxonomic units of bacteria that do not contain recognizable sequences of any of the known l-asparaginase types, implying that those microorganisms most likely contain new, as yet unknown types of l-asparaginases. Such novel enzymes, when properly identified and characterized, could hold promise as antileukemic drugs.

## Introduction

Ammonia, NH_3_, as a source of nitrogen, is undoubtedly one of the fundamental chemical ingredients found at the beginning of many metabolic pathways. It is thus understandable that nature has evolved enzymes that hydrolyze primary amides, such as at the side chain of l-asparagine, to produce ammonia. However, the reason why three large and completely unrelated Classes of l-asparaginases (EC 3.5.1.1) have evolved to carry out this simple reaction does not have yet a clear explanation.

Historically, enzymes in these three Classes (1, 2, 3) were named after the organism in which they were first found^1,2^, leading to an awkward classification into bacterial-, plant- and *Rhizobium etli*-type l-asparaginases^3,4^. The old nomenclature was confusing because members of all three Classes are distributed over all domains of life. Classes 1 and 3 are further subdivided into types. Specifically, in Class 1, cytosolic type I (AI) enzymes with low (mM) substrate affinity^5^, and periplasmic type II (AII) enzymes with much higher (μM) substrate affinity^6^ are present. In *E. coli*, EcAI is constitutively produced, while the expression of EcAII is induced in anaerobic conditions^7^. The cytoplasmic *E. coli* EcAIII enzyme from Class 2 (and type III, AIII), with dual l-asparaginase/isoaspartyl aminopeptidase activity, is responsible for the degradation of not only l-asparagine (with mM affinity) but also of the harmful isoaspartyl peptides^8^. EcAIII is an Ntn-hydrolase and develops its enzymatic activity upon autoproteolytic maturation into subunits α and β^9^. In Class 3, we have constitutive type AIV enzymes, exemplified by the *Rhizobium etli* protein ReAIV, and induced type AV enzymes, exemplified by *R. etli* ReAV^10^. ReAV is induced by l-Asn^11^ and shows a visible allosteric effect and low mM substrate affinity at high pH. Surprisingly, the tightly bound zinc cation, with its open coordination sphere (complemented by a water molecule), is not required for the l-asparaginase activity of ReAV^10^.

l-Asparaginases from different Classes show distinct architecture and oligomeric states. Class 1 enzymes, e.g., EcAI and EcAII (Fig. S1a,b), are generally homotetramers (dimers of two intimate dimers)^12^. Some type I enzymes (e.g., from *Pyrococcus horikoshii*) have been reported, however, to function as (intimate) dimers^13^ or as hexamers (e.g., from *Thermus thermophilus*)^14^. Each protomer in this Class is built of two domains, the N-terminal and C-terminal domain. In Class 1 proteins, e.g., in EcAII, the active site with the nucleophilic threonine is in the N-terminal domain and its structure is completed upon binding the l-Asn substrate, which induces the closure of a flexible gating element^15,16^. The architecture of EcAI is similar but stabilizing the substrate in the active site of one protomer requires the involvement of residues from the C-terminal domain of its intimate partner. EcAI is allosteric, meaning that except for the active site there is another (allosteric) binding site for l-Asn^5^. The architecture of Class 2 enzymes is entirely different—the immature precursor of EcAIII (Fig. S1c) is a homodimer with sandwich-like topology and with the mature chains α and β of each subunit connected by a linker. In the autocleavage process, a catalytic threonine is liberated at the N terminus of subunit β and the linker region becomes disordered or even partially degraded^9^. Enzymes from Class 3 possess yet another architecture, not related to the structures of enzymes from Class 1 or 2 (Fig. S1d,e), but more similar to serine β-lactamases or penicillin binding proteins (PBPs). The only known structure of a Class 3 enzyme has been recently published^10^ for the archetypical ReAV protein from *R. etli*. ReAV is a homodimer of two subunits, each built of two domains: the catalytic and dimer stabilization domain. In contrast to Class 1 and Class 2 enzymes, ReAV binds a zinc cation in an unusual 2xCys/Lys/water coordination pattern^10^.

The grouping of l-asparaginases into three Classes (bacterial, plant, and rhizobial) seems to correspond well with their evolutionary relatedness. Based on the presence of the catalytic domains, sequence similarity, and structural properties, it is obvious that types I and II are most closely related to each other. Similarly, types IV and V share common properties, suggesting their recent common origin. On the other hand, type III clearly represents the most distinct group of all l-asparaginases.

The subject of the present analysis is the molecular evolution of enzymes from the five types and three Classes of l-asparaginases in the Bacteria domain. In particular, we were interested in how these enzymes are distributed in different groups of bacteria. It is also interesting to know how these five l-asparaginase enzymes have been shuffled in and out between different taxonomic groups. Using the full collection of bacterial l-asparaginases we have asked a question about their sequence conservation, including variability in the highly conserved functional domains.

Studying l-asparaginases is important not only from the point of view of phylogeny and evolution. Bacterial (Class 1) l-asparaginase of type II (e.g., EcAII) have usually sufficiently high (μM) substrate affinity to make them successful drugs in the treatment of acute lymphoblastic leukemia (ALL). The administration of these bacterial proteins (e.g., Elspar from *E. coli* or Erwinase from *Erwinia chrystanthemi*) is not without adverse side effects, which sometimes preclude successful outcome^17^. New antileukemic l-asparaginases are thus urgently needed. One way of approach to this issue has been enzyme engineering aiming at the conversion of alternative types of l-asparaginases into high-affinity enzymes^18^. Unfortunately, this approach has not been successful thus far. On the other hand, one might expect that in the huge enzyme engineering experiment carried out by Nature on the evolutionary scale, solutions to this medicinal problem may have been found already. With this motivation, we present a pan-genomic analysis towards the identification of promising natural variants of the currently used enzymes, or of entirely new bacterial l-asparaginases that might meet the criteria as candidate antileukemics.

## Results and discussion

### Over a quarter of bacterial species lack detectable sequence marks of known l-asparaginases

To explore the distribution of l-asparaginase genes among Bacteria, we used a reference data set of the four originally identified bacterial enzymes (EcAI, EcAII, ReAIV, ReAV) and the *E. coli* ortholog (EcAIII) of plant l-asparaginases (Supporting Information) as the prototypic representatives of types I, II, IV, V, and III, respectively. The corresponding proteins are designated as AI, AII, AIV, AV, AIII, while their coding genes as *aI*, *aII*, *aIV, aV, aIII*. In agreement with l-asparaginase classification^2^, these five proteins represent the three structural Classes, highlighted by different assignments in the Pfam database^19^. Accordingly, EcAI and EcAII belong to the l-asparaginase PF00710 family characterized by the presence of N-terminal and C-terminal l-asparaginase domains, EcAIII has a single domain belonging to the PF01112 family, while ReAIV and ReAV contain an l-asparaginase domain from the PF06089 family (Fig. S2). Despite the similarity of protein domain content, sequence identity within the EcAI/EcAII and ReAIV/ReAV pairs is low, 23.6% and 30.7%, respectively.

To identify orthologs of the five types of l-asparaginases in Bacteria, we searched genomic and protein sequences of 45,555 bacterial species (255,090 genomes) from the Genome Taxonomy Database (GTDB)^20,21^. Specifically, we screened protein sequences for the presence of any of the Pfam l-asparaginase domains (i.e., PF00710, PF01112, PF06089) and assigned each protein to one of the five l-asparaginase types according to the highest sequence similarity (see “Methods”). We found at least one type of l-asparaginase in 72% (*n* = 32,940) of the bacterial species. In the remaining 28% of the bacterial species (*n* = 12,615), the protein sequences did not contain any known l-asparaginase domains and lacked sequence similarity that would allow orthologous assignment (“Methods”) to any of the five l-asparaginase types. Although genome assemblies of species without detectable l-asparaginases had significantly lower quality in terms of completeness, sequence continuity as well as protein and tRNA content (*P* < 10^–5^; Mann–Whitney U-test) than genomes with at least one type of l-asparaginase (Fig. S3), it is unlikely that low genome quality can explain all the missing l-asparaginase genes in whole taxonomic units of bacteria that are represented by multiple genomes of variable sequence quality. Specifically, we observed almost one thousand of such taxonomic units (across all taxonomic levels from species to phylum) that do not contain recognizable sequence features of any of the five l-asparaginase types (Table S1). The prime examples are two families of obligate intracellular bacterial species, namely the *Anaplasmataceae* (from Proteobacteria phylum) and *Chlamydiaceae* (from Chlamydiota phylum) families that contain more than 50 species and are represented by more than 1,500 genome assemblies. We conclude that these genomes either do not encode l-asparaginases of the five known types, or the corresponding protein sequences lack sufficient similarity and recognizable asparaginase-related domains and thus escaped our detection.

Since l-asparaginase is an obligatory activity for cell viability, identification of bacterial taxonomic units with no detectable marks of this enzyme strongly suggests the existence of some other, so far undiscovered proteins with l-asparaginase activity, at least in the Bacteria domain. Although no obvious taxonomic and phylogenetic pattern emerges after analyzing genomes without l-asparaginases AI-AV, we observe that 94% of them do not contain recognizable glutaminase domains as well.

### l-Asparaginase of type I is the most abundant in bacterial species

**Figure 1:**
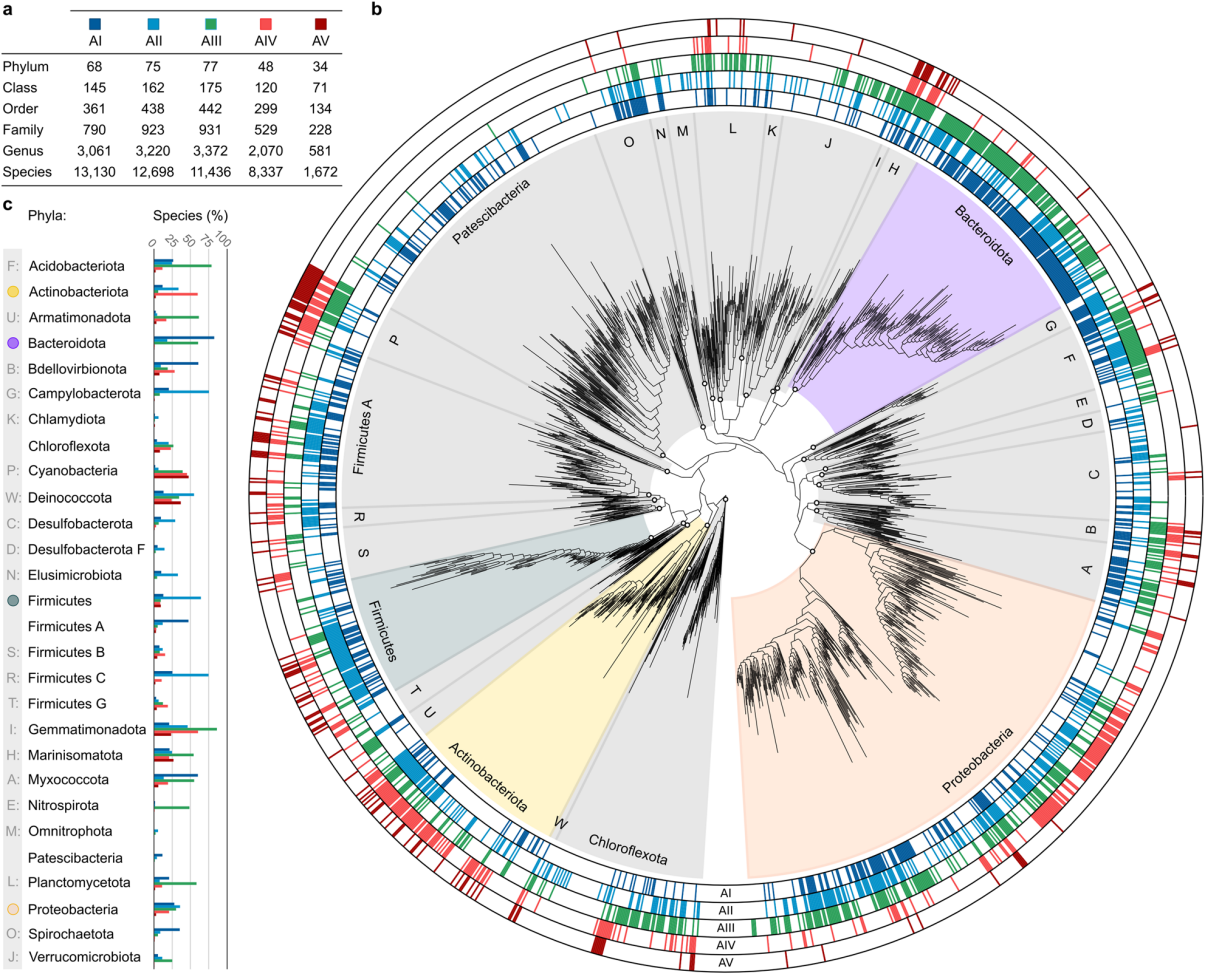
Abundance of l-asparaginases in bacteria. (**a)** The table shows the number of taxonomic groups of bacteria containing each of the five l-asparaginase types (i.e., AI, AII, AIII, AIV, and AV). For example, AI l-asparaginase is present in 13,130 species belonging to 3061 genera and 790 families. (**b)** The phylogenetic tree of Bacteria illustrates the presence or absence of the five types of l-asparaginases in the phylogenetic taxa. The tree encompasses 1665 bacterial families belonging to 28 most abundant phyla and covering 96% of all bacterial species. Clades of the four largest bacterial phyla (Proteobacteria, Actinobacteriota, Bacteroidota, and Firmicutes) cover 75% of species and are highlighted in the tree. The five outer rings provide information about the presence (filled with color) or absence (white) of each l-asparaginase type in a bacterial family. (**c)** List of the phyla shown in the tree and the percentage of species in each phylum containing enzyme types AI, AII, AIII, AIV, and AV.

We investigated the distribution of the five types of l-asparaginases across all taxonomic units in the Bacteria domain (Fig. 1, Table S2). l-Asparaginase of type I (AI) is present in the highest number of bacterial species (*n* = 13,130), followed by AII, AIII, AIV, and AV (Fig. 1a). Interestingly, both AII and AIII enzymes have a broader phylogenetic range than AI, spanning a larger number of phyla (Fig. 1a). The *Rhizobium etli*-types, AIV and AV, extend far beyond the *Rhizobium* genus, spanning a majority of bacterial phyla except four, namely Patescibacteria, Desulfobacterota clade F, Elusimicrobiota, and Omnitrophota (Fig. 1b,c). Among the four largest bacterial phyla (Proteobacteria, Actinobacteriota, Bacteroidota, and Firmicutes), which cover 75% of all bacterial species, AI and AIII are predominantly found in Bacteroidota, AII is most frequent in Firmicutes, and AIV is most common in Actinobacteriota (Fig. 1b,c). Although AV is the least abundant l-asparaginase in Bacteria, present in five times fewer species (*n* = 1672) than AIV (*n* = 8337), the protein is the most common l-asparaginase type in Cyanobacteria, and together with AIV is found in almost half of all Cyanobacteria species (Fig. 1c).

Although the five l-asparaginase types are present in almost all bacterial phyla, in some cases their distribution varies in different taxonomic units (Fig. 1b). To identify taxonomic groups with l-asparaginase over- and underrepresentation, we performed an enrichment analysis of each l-asparaginase type across all taxonomic units higher than species (see “Methods”). In most taxa (92%), all five l-asparaginase types are present in accordance (*P* > 10^–5^) with their expected distributions. In the remaining 8% of taxa, at least one type of l-asparaginase is significantly over- or underrepresented (*P* < 10^–5^) (Tables S3–S4).

As expected, the bacterial-type l-asparaginase AI shows significant overrepresentation (*P* = 5.14 × 10^–172^) in the *Enterobacteriaceae* family (phylum Proteobacteria) by covering nearly 80% species (*n* = 572; including *Escherichia fergusonii* and *Shigella flexneri*). However, the protein has even higher representation (*P* ≈ 0) in the family *Flavobacteriaceae* (phylum Bacteroidota), where it is found in 87% of species (*n* = 1089). Similarly, bacteria from the *Flavobacteriaceae* family are enriched in the plant-type l-asparaginase AIII (*P* ≈ 0), which is present in 83% of its species. Type II l-asparaginases show the highest overrepresentation (*P* ≈ 0) in the *Burkholderiaceae* family (phylum Proteobacteria) where they are present in 92% of all species (*n* = 1528).

The *R. etli*-type l-asparaginase AIV is characteristic of the *Rhizobiaceae* family (phylum Proteobacteria), where the protein is present in almost all species (99% of *n* = 608; *P* ≈ 0). In addition, the type IV enzyme is also highly overrepresented in three families belonging to the phylum Actinobacteriota, namely *Micrococcaceae*, *Micromonosporaceae* and *Streptosporangiaceae*, where it is present in 100% (*n* = 310), 97% (*n* = 200), and 96% (*n* = 212) of all species, respectively. Intriguingly, the type V l-asparaginase shows neither over- nor underrepresentation (*P* = 0.5) in the *Rhizobiacea* family and occurs only in 4% of the species in this family. This protein is the most overrepresented (P < 1.0 × 10^–110^) in *Paenibacillaceae* (phylum Firmicutes), *Cyanobiaceae* (phylum Cyanobacteria), and *Burkholderiaceae* (phylum Proteobacteria).

### Many bacterial genomes encode either AI + AIII or AII l-asparaginases

Analysis of co-occurrence of different types of l-asparaginases showed that most often a bacterial genome encodes only one type, except for AIII, which is most frequently accompanied by an AI enzyme (Table 1).

**Table 1:** Co-occurrence of five l-asparaginase types in the genomes of 32,940 bacterial species.

					Phylum	Class	Order	Family	Genus	Species
AI					51 (68)	108 (145)	264 (361)	527 (790)	1692 (3061)	6292 (13,130)
	AII				65 (75)	126 (162)	303 (438)	570 (923)	1613 (3220)	5652 (12,698)
			AIV		41 (48)	91 (120)	206 (299)	351 (529)	1287 (2070)	4908 (8337)
AI		AIII			32 (33)	63 (70)	129 (151)	274 (330)	994 (1239)	3838 (4837)
		AIII			70 (77)	144 (175)	297 (442)	529 (931)	1207 (3373)	2947 (11,436)
	AII	AIII			35 (40)	60 (71)	150 (188)	258 (369)	767 (1165)	2146 (3467)
	AII		AIV		29 (32)	41 (53)	90 (116)	153 (192)	490 (620)	1649 (2066)
AI	AII				26 (33)	43 (61)	88 (120)	179 (248)	355 (640)	1494 (2430)
AI	AII	AIII			14 (15)	25 (27)	41 (45)	97 (101)	283 (298)	778 (845)
		AIII	AIV		24 (27)	37 (44)	79 (108)	111 (157)	322 (484)	748 (1188)
				AV	28 (34)	48 (71)	77 (134)	111 (228)	203 (581)	536 (1672)
AI			AIV		18 (21)	29 (38)	52 (75)	75 (114)	151 (251)	339 (585)
	AII			AV	20 (21)	25 (31)	42 (58)	55 (84)	107 (175)	286 (632)
		AIII		AV	11 (16)	15 (27)	33 (51)	57 (84)	148 (207)	271 (560)
	AII	AIII	AIV		16 (17)	20 (21)	38 (42)	53 (60)	123 (142)	248 (297)
	AII	AIII		AV	12 (12)	13 (15)	20 (23)	24 (28)	41 (47)	220 (254)
AI		AIII	AIV		13 (14)	17 (19)	27 (31)	36 (42)	71 (88)	130 (173)
			AIV	AV	9 (16)	16 (26)	27 (47)	30 (58)	58 (107)	121 (216)
AI				AV	11 (16)	14 (23)	22 (36)	26 (43)	45 (79)	65 (160)
	AII		AIV	AV	6 (6)	8 (8)	16 (16)	20 (21)	35 (41)	59 (70)
AI	AII		AIV		12 (14)	15 (18)	21 (27)	26 (33)	38 (58)	58 (102)
AI	AII	AIII	AIV		6 (6)	7 (7)	11 (11)	12 (12)	21 (21)	41 (41)
AI	AII			AV	5 (7)	5 (7)	8 (10)	8 (10)	13 (18)	30 (59)
AI	AII	AIII		AV	3 (3)	3 (3)	3 (3)	3 (3)	4 (4)	26 (26)
AI		AIII		AV	9 (10)	11 (12)	12 (14)	14 (16)	16 (20)	22 (50)
AI			AIV	AV	6 (7)	6 (7)	8 (9)	8 (9)	9 (13)	12 (17)
		AIII	AIV	AV	7 (9)	7 (11)	9 (13)	9 (14)	11 (18)	11 (21)
	AII	AIII	AIV	AV	2 (2)	3 (3)	3 (3)	4 (4)	6 (6)	8 (8)
AI	AII		AIV	AV	1 (1)	1 (1)	1 (1)	1 (1)	3 (3)	3 (3)
AI		AIII	AIV	AV	2 (2)	2 (2)	2 (2)	2 (2)	2 (2)	2 (2)

The table rows show the exact combination of the different l-asparaginase types and information on the number of taxonomic units in which the combination occurs. Numbers in parentheses indicate taxonomic units with at least all types in each combination. Combinations of l-asparaginases are ordered according to the decreasing number of species.

When considering combinations of l-asparaginase types, AI occurs together with AIII in bacterial genomes twice as often as with AII. Moreover, the two *E. coli*-type enzymes (AI and AII) are simultaneously present (separately or in various combinations with other types; Table 1) not only in *Escherichia* relatives but also in over one thousand other, distantly-related species belonging to 32 phyla other than Proteobacteria, including Actinobacteriota, Bacteroidota, and Firmicutes.

Although the *R. etli* genome encodes both, ReAIV and ReAV, the AIV and AV proteins are rarely present together in other bacteria. While AIV is present in almost all *Rhizobium* species (*n* = 132 out of 133), AV is found in *Rhizobium* species six times less frequently (*n* = 23) (Table S5). In general, genomes of only 216 bacteria species (0.5%) have both AIV and AV genes, among which 121 species contain exclusively only these two types of l-asparaginases. AIV is more frequently found together with AII, AIII and AI than with AV, and conversely, AV co-occurs more frequently with AII and AIII rather than with AIV (Table 1).

We also identified several taxonomic groups that show statistically significant underrepresentation of at least one l-asparaginase type (Table S4). Both AI and AIII enzymes show the highest underrepresentation in the Actinobacteriota phylum (*P* ≈ 0). The deficit of the AI and AIII proteins in Actinobacteriota seems to be compensated by overrepresentation of type AII (P = 1.6 × 10^–16^) and type AIV (*P* ≈ 0) proteins in this phylum. This result is also supported by the observation that a higher representation of the AI and AIII proteins in the *Enterobacteriaceae* family coincides with the underrepresentation of AII l-asparaginase (*P* = 4.5 × 10^–33^; Table S4). The *R. etli*-type proteins AIV and AV are the most underrepresented in the Bacteroidota phylum (*P* ≈ 0). This deficit, on the other hand, seems to be compensated by the higher representation of the AI and AII proteins (*P* ≈ 0).

We did not find a genome containing the complete repertoire of the five l-asparaginase genes among 255,090 bacterial strains. However, combinations of four types of l-asparaginases can be observed very sporadically in eight bacterial phyla, including Proteobacteria, Actinobacteriota, Bacteroidota, and Firmicutes. The most common combination of four l-asparaginase types, AI + AII + AIII + AIV, was found in six phyla and 41 species (Table 1), including one *Rhizobium* species (Table S5). A similar combination of four l-asparaginases, AI + AII + AIII + AV, was found predominantly in Burkholderiales species belonging to Proteobacteria. Other combinations of four l-asparaginases (Table 1; last three rows) were observed in a total of 13 species including *Rhizobium bangladeshense* (AII + AIII + AIV + AV).

Taken together, the distribution of the l-asparaginase types shows an interesting pattern when analyzed at the l-asparaginase Class level. When looking at the global co-occurrence of the l-asparaginase types (Table 1), we observe a preference for genomes to contain pairwise combinations of enzymes belonging to distinct Classes (e.g., AI and AIII, AII and AIII, or AII and AIV), with frequencies closely following the abundances of single-gene-containing species. Interestingly, this observation also holds for three l-asparaginase types containing species, the most frequent combination being AII + AIII + AIV (Table 1). Such a distribution may suggest that instead of accumulating genes encoding enzymes with similar properties (belonging to the same Class), in most cases bacteria prefer to expand their repertoire of available l-asparaginases by proteins representing distinct structural and biochemical properties. This strategy may lead to an expansion of the biological capacity of the species and provide selective advantages. Moreover, the evident preference for single-asparaginase-containing genomes and the decreasing fraction of species with a higher number of l-asparaginases may suggest that increasing the number of enzyme types does not offer any general selective advantage.

### Horizontal gene transfer can explain the puzzling distribution of most l-asparaginase genes

l-Asparaginase genes representing a particular type are present in bacterial genomes mostly as single copies, suggesting that gene duplication is not a leading mechanism of their expansion. In particular, the *aI*, *aIV*, and *aV* genes are present as single copies in more than 90% of bacterial species, and the *aII* and *aIII* genes also lack paralogs in 85% and 70% of species, respectively (Table S6). Although among the five l-asparaginase types, gene duplication is most frequent for the *aIII* gene, which is present in two copies in the genomes of almost one-quarter species, *a*II can be found in a higher number of copies per genome than *aIII*. For example, eight species of *Burkholderiaceae* contain six, seven, or even eight *aII* copies (Table S7). Moreover, we observe a weak negative correlation in the number of gene copies between *aI* and *aII* (Spearman’s rho = −0.33; P ≈ 0) as well as between *aI* and *aIV* (rho = −0.39; P ≈ 0), further supporting the conclusion that the absence of one of the types could be compensated by the presence (of additional copy/copies) of another type (Table S8).

**Figure 2:**
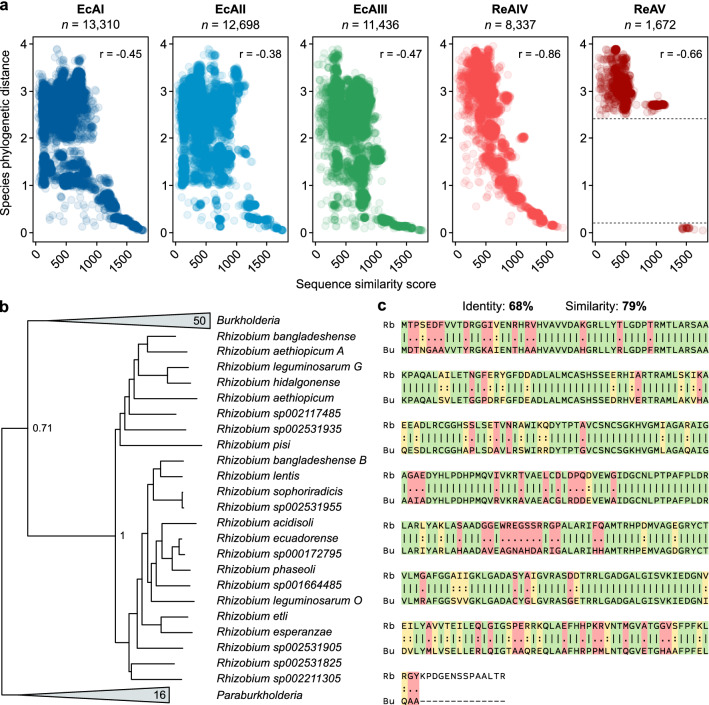
Relation between sequence similarity of l-asparaginase proteins and phylogenetic distance between species. **(a)** Alignment score of orthologous l-asparaginases and phylogenetic distance separating the bacterial species. Protein sequence of each prototypic enzyme (EcAI, EcAII, EcAIII, ReAIV, and ReAV) was separately aligned to all its orthologous sequences from other bacterial species. The phylogenetic distance between bacterial species was obtained from the GTDB reference tree of bacteria. Each dot in the scatterplots represents a single comparison between a prototypic enzyme protein and an orthologous sequence from other bacterial species. (**b)** Fragment of the phylogenetic tree of AV proteins in bacterial species (*n* = 1672) showing close evolutionary relation of AV proteins between the species of *Rhizobium* (*n* = 23) and *Burkholderia* (*n* = 50). Bootstrap support values are shown on the main tree branching. (**c**) Global sequence alignment of AV proteins from *Rhizobium bangladeshe* (Rb) and *Burkholderia ubonensis* (Bu), with sequence identities (green), similarities (yellow), and differences (red) highlighted*.*

To further investigate the evolutionary pathways that have led to the current distribution of l-asparaginase genes among bacteria, we performed pairwise amino-acid sequence alignments between each reference protein (EcAI, EcAII, EcAII, ReIV, and ReAV) and all its orthologs. The resulting sequence similarity scores were then plotted against the phylogenetic distance separating the bacterial species of the aligned sequences (Fig. 2a).

The level of similarity of l-asparaginase sequences is generally correlated with the phylogenetic distance separating the host bacterial species. The sequence similarity of the *E. coli*-type l-asparaginases (EcAI, EcAII, EcAIII) shows a moderate negative correlation with species distance (Pearson’s *r* between −0.38 and −0.47). The highest absolute correlation was observed for the ReAIV protein (Pearson’s *r* = −0.86), suggesting that the rate of change in the AIV sequence follows the rate of evolution of the genome of the global species.

Interestingly, we observed that the ReAV protein from *R. etli* does not have orthologs in closely related bacteria but shows the highest sequence identity to AV proteins in the distant species of *Burkholderia*. This close evolutionary relation of *aV* genes between *Rhizobium* and *Burkholderia* species is also supported phylogenetically (Fig. 2b), suggesting horizontal gene transfer (HGT) between *Rhizobium* and *Burkholderia* species. Despite the large evolutionary distance between the genera (different classes of Proteobacteria), the median sequence identity and similarity between their AV proteins (63% and 75%, respectively) are nearly twice as high as the median identity and similarity of AV orthologs between all bacteria (33% and 42%, respectively; Fig. S4). We observe the highest sequence identity of the AV proteins between *Rhizobium bangladeshe* and *Burkholderia ubunensis*, suggesting that the HTG event occurred between these species (Fig. 2c). Given that the AV proteins are found more frequently in *Burkholderia* species (50 out of 62) than in *Rhizobium* species (23 out of 133), *Burkholderia ubunensis* could be considered as a probable donor of the *aV* gene (Fig. 2b). This hypothesis about HGT origin of rhizobial AV is additionally supported by reports indicating that *Rhizobium* and *Burkholderia* species often coexist as root nodule symbionts of various legume plants^22–24^.

**Figure 3:**
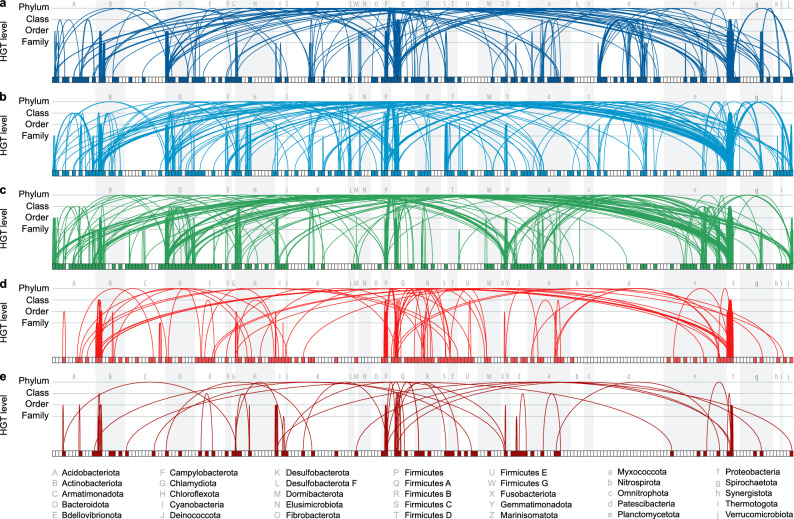
Putative horizontal gene transfer events of l-asparaginases in bacteria. HGT events of the five AI-AV l-asparaginase types (**a–e**, respectively) across 223 classes of bacteria belonging to 35 most abundant phyla. Rectangles represent classes of bacteria and mark the presence (filled with color) or absence (white) of a given l-asparaginase type in a given class. Arcs show horizontal gene transfer between two bacterial species. The height of the arcs marks the highest taxonomic rank that is different between the species (i.e., phylum, class, order, family). Arc widths are arbitrary and do not represent any taxonomic or evolutionary distance between bacteria.

We further explored the putative HGT events in the evolution of all five types of l-asparaginase genes by reconstructing phylogenetic trees for all types and comparing them with the reference species tree. Specifically, we manually examined the evolutionary histories of l-asparaginases and species to detect conflicting phylogenies analogous to the case of the *aV* gene in *Rhizobium* and *Burkholderia*. We identified 1,795 potential HGT events among all five l-asparaginase types (Table S9). Most of the HGTs (92%) affected bacterial species from 35 of the most abundant phyla (Fig. 3). The highest number of species involved in HGT was recorded for the *aIII* gene (9.8%), followed by *aII* (8.5%) and *aV* (8.0%). *aI* and *aIV* were less prone to HGT, with, respectively, 4.7% and 5.5% of bacterial species affected. The mean protein sequence identity of potentially horizontally transferred l-asparaginase genes (55–66%) was twice as high as for orthologous l-asparaginases in general (P < 8.7 × 10^–5^; two-sample t-test), supporting the existence of a recent common ancestor of these l-asparaginase proteins encoded in genomes of distant species. In addition, among the characterized HGT events, we found 24 pairs of taxonomic units between which more than one type of l-asparaginase was transferred (Table S10). The highest exchange rate, involving three types of l-asparaginase, occurred between bacteria from two taxonomic pairs, namely between *Burkholderiaceae* and *Pseudomonas clade E* (AII, AIII, AV) as well as between *Pseudonocardiaceae* and *Streptosporangiaceae* (AII, AIII, AIV).

We also identified more than 300 single taxonomic units involved in multiple independent HGT events of different l-asparaginase types (Table S11). Interestingly, five taxonomic groups were implicated in HGT events that involved all five types of l-asparaginases. These taxonomic groups include three families (*Burkholderiaceae*, *UBA6960*, and *Clostridiaceae*) and two genera (*Pseudomonas clade E* and *Neobacillus*).

Altogether, the scattered distribution of l-asparaginases among bacteria is in part a result of horizontal gene transfer between distantly related bacteria. It must be noted that the applied procedure probably missed HGT events occurring between closely related species. Hence, our data show only the most prominent examples, while the real extent of HGT affecting the distribution of l-asparaginase genes among bacterial species is most probably much more salient. On the other hand, our HGT investigations were restricted to the sequences representing only bacteria. We cannot, therefore, trace horizontal transfer events that occurred between more distant organisms, e.g., bacteria and eukaryotes, as already reported for ReAV^25^.

### Sequence variability of l-asparaginases is restricted to peripheral solvent-exposed regions

**Figure 4:**
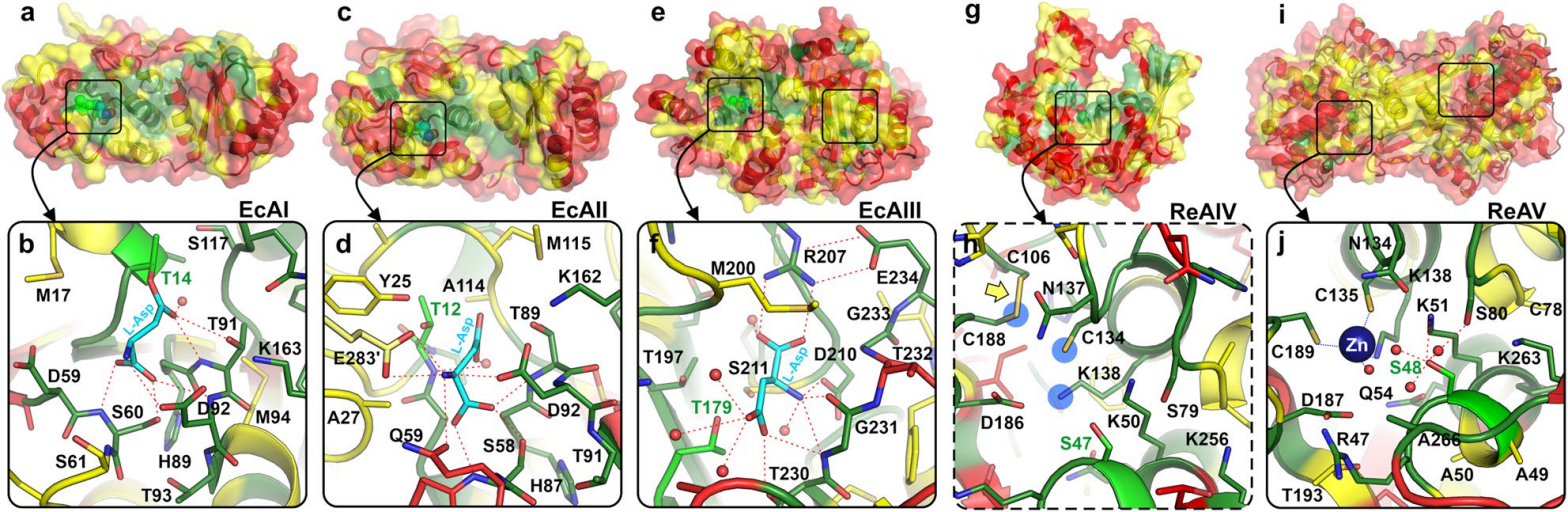
Conservation of residues and the active sites of representative l-asparaginases. Residues are colored according to their conservation: red (highly variable: 0–30% identity), green (highly conserved, 80–100% identity), or yellow (30–80% identity). (**a**,**b)** The EcAI subunit A **(a)** and a covalent reaction intermediate **(b)** with a substrate molecule (cyan) in the active site (PDB ID: 2him). (**c,d)** The EcAII subunit A with the l-Asp product (cyan) bound in the active site (PDB ID: 3eca). (**e,f)** The EcAIII (ɑ + β)_2_ homodimer with the l-Asp product (cyan) bound in the active site (PDB ID: 2zal). (**g,h)** A protomer of ReAIV predicted by the Robetta server with a detailed view **(h)** of the residues in the putative active site; residues potentially involved in Zn^2+^ coordination are marked by blue circles; the predicted S–S bridge between Cys188 (putative metal coordination ligand) and Cys106 that might be formed in the absence of a metal cation is marked by a yellow arrow. (**i,j)** The ReAV homodimer **(i)** and the active site **(j)** with the Zn^2+^ ion (dark blue sphere) coordinated close to the nucleophilic Ser48 (PDB ID: 7os5). In all panels, the nucleophilic residue (Thr or Ser) is conserved and colored light green.

For each of the five l-asparaginase types, we mapped the level of sequence conservation within all orthologs on the 3D structure of the prototypical protein (Fig. 4). For EcAI, EcAII, EcAIII and ReAV, the available crystal structures were retrieved from the PDB. Since ReAIV has no experimental structure model, we used AlphaFold2^26^ and Robetta^27^ for structure prediction.

Analysis of the distribution of the conservative regions in the sequence of EcAI revealed that the lowest sequence variability is observed in the active site, its close neighborhood, and the region extending to the allosteric site. Residue conservation at other structural elements important for enzyme action, such as the dimer interface and linker connecting the N- and C-terminal domains, is rather low (Fig. 4a,b). A similar distribution of conserved and variable residues is observed in the case of EcAII; however, this enzyme does not have an allosteric site (Fig. 4c,d). The active site of the Ntn-hydrolase EcAIII is also the most highly conserved part of the structure. Conserved residues also appear in the β-strands located in the protein core, in the close vicinity of the active site (Fig. 4e,f). The same trend is observed in the structures of ReAV and its predicted structural homolog ReAIV (Fig. 4g–j); sequence variability is very low in the region of the active site (including the metal coordination sphere) located in the center of the molecule, while far from the protein interior, sequence conservation is visibly lower.

As expected, strictly conserved residues are found in the active sites, consistent with the specific geometry of the catalytic apparatus required for selective binding and hydrolysis of l-asparagine, while low level of conservation is seen in variable structural elements, such as domain linkers or loops, generally located on the surface of the protein molecules. This observation lends a powerful cross-validation aspect to our study, supporting on the one hand the correctness of the grouping of the diverse proteins, and on the other confirming the active site composition and location in newly detected l-asparaginases.

### Sequence variability within the functional domain of AII l-asparaginases points to enzymes with possible new catalytic properties

l-Asparaginase orthologs identified in this study span the whole Bacteria domain and thus provide a unique resource for exploring their sequence properties in the context of enzymatic activity. We focused on type II enzymes, as their representatives from *Escherichia* and *Erwinia* are clinically used to treat ALL. However, these therapeutic enzymes also show toxicity, which can be attributed, *inter alia*, to their l-glutaminase co-activity^28^.

**Figure 5:**
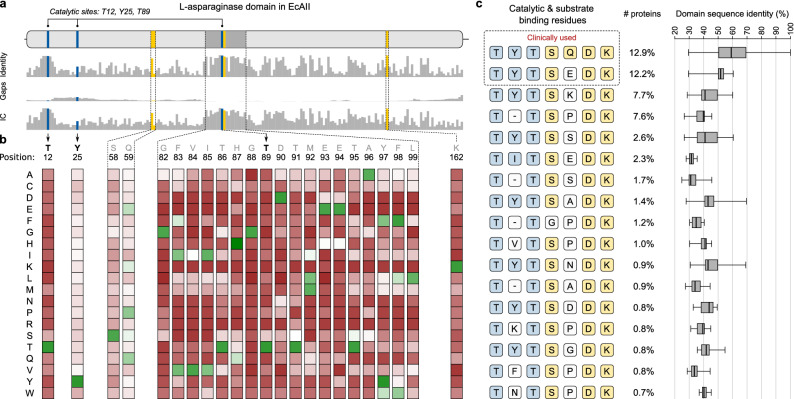
Sequence characteristics of l-asparaginase domain in bacterial AII proteins. **(a)** Amino acid conservation along the l-asparaginase domain between orthologs and the reference EcAII protein. The catalytic residues are marked by blue bars and substrate-binding residues are shown in yellow. The bar charts show identity percentage, gaps percentage and information content (IC) at each site. (**b)** Position-specific score matrix (PSSM) calculated with reference to EcAII, showing how often a given residue is found at a specific position within the domain. Preferred residues (occurring more often than their expected frequency) are shown in green and avoided amino acids (occurring less often than their expected frequency) are shown in red. The complete PSSM profile across all l-asparaginase domain sites is shown in Table S12.** (c)** Most common arrangements of structurally and functionally important amino acid residues—catalytic (blue) and substrate binding (yellow)—found in bacterial AII proteins. The first two residue patterns are present in clinical drugs used to treat ALL (*E. coli strain K12* and *Erwinia chrysanthemi*). The percentage numbers indicate the fraction of AII proteins containing a given residue pattern. Box plots (on the right) show sequence identity distribution of the full-length AII l-asparaginase domain across orthologs containing a given residue pattern. The box plot with sequence identity statistics of the whole l-asparaginase domain may be interpreted as a proxy of a dispersal range of a given motif among diverse bacteria.

The N-terminal domain of EcAII contains regions that are functionally important for the l-asparaginase activity. We created a sequence profile of the l-asparaginase domain sequences to identify conserved and variable amino acid residues at each domain site (Fig. 5a,b). We do not observe any preferable substitutions (occurring more often than expected by chance) of the catalytic site residues—Thr12, Tyr25, Thr89—indicating their functional importance. The two threonines are preserved in 96% and 95% of orthologs, respectively; Fig. 5a,b, and in the remaining orthologs are most often substituted by Ser. Although Tyr25 is conserved in only half of the orthologs (48%), none of the observed amino acid substitutions is preferred since they occur less frequently than by chance (Fig. 5b). Among the substrate binding residues—Ser58, Gln59, Thr89, Asp90 and Lys126—glutamine at site 59 is the least conserved (14% orthologs) and is preferentially substituted with Glu, Lys, or Pro. Of note, the therapeutic enzyme from *E. coli* has Gln59 and the *Erwinia* enzyme has Glu59 (Fig. 5c). It was reported previously that in type II enzymes, the residue at EcAII position 59 (together with those at positions 248 and 283) determines the affinity for l-glutamine. Enzymes with negligible glutaminase co-activity have Gln at position 59^5,29^, while those with significant glutaminase co-activity have Glu at position 59. Considering these known dependencies, our sequence profile analysis can facilitate future search for potential therapeutic enzymes with desired substrate specificity.

The sequence profile of the AII l-asparaginase domain (Fig. 5b) assumes independence between positions, however it can be expected that the most conserved arrangements of residues (i.e., present in a large number of orthologs) should preserve the enzymatic activity. We, therefore, looked at all combinations of the catalytic (Thr12, Tyr25, Thr89) and substrate-binding (Ser58, Gln59, Thr89, Asp90 Lys126) residues in bacterial AII proteins (Fig. 5c). Two such amino acid patterns found in the enzymes from *E. coli* and *E. chrysanthemi* are most commonly present in bacteria (in 13% and 12% species, respectively) (Fig. 5c). Notably, the proteins containing these two combinations of residues show a wide range of sequence identity of the full-length l-asparaginase domain (median sequence identity of 58% and 52%, respectively; Fig. 5c right panel). Two other common residue combinations, not found in currently used therapeutic l-asparaginases (“TYTSKDK” and “T-TSPDK” in the left panel of Fig. 5c), are present in more than 7% of species each (median sequence identity of 42% and 39%) and spread across 16 bacterial phyla. Such high conservation of two alternative residue combinations in 1588 proteins (Table S13) that are evolutionary distant to commercially used AII proteins can markedly affect the AII protein structure, l-asparaginase activity, and immunogenicity. Although the experimental screening techniques for a lead compound for AII-based drug candidates are still expensive and time-consuming, subsequent in silico studies may further narrow down the candidate proteins to a smaller set of best candidates for experimental verification. For example, molecular modeling and docking have proven suitable for studies involving screening for alternative l-asparaginase candidates and enzyme optimization^30^. Docking asparagine to the AII structures predicted by homology modeling was used in screening for l-asparaginase enzymes with reduced glutaminase activity^31^. Those studies were also validated using in vitro experiments on the identified candidates^32^.

In summary, the variability of the highly conserved functional regions of type II l-asparaginases showed patterns of residue arrangements that probably correlate with the enzymatic properties of the proteins. This finding is especially interesting in the context of the therapeutic applications of the AII enzymes. The newly found arrangements of the crucial catalytic residues are present in concrete living organisms and, therefore, probably represent their adaptation to specific environmental conditions. This adaptive natural selection may have produced functional l-asparaginases that possess catalytic features that are interesting from the point of view of medicinal applications.

## Material and methods

### Sequence data and taxonomy of bacteria

Sequence data, taxonomic affiliations, and phylogenetic tree of Bacteria were retrieved from the Genome Taxonomy Database (GTDB) release 06-RS202 (April 2021)^20,21^. The sequence data contained 254,090 genomes and 45,555 proteomes of 45,555 bacterial species. For the remaining 208,535 genomes, protein sequences were predicted using Prodigal v2.6.3^33^, yielding in total 931,246,625 protein sequences. The reference tree of bacterial species obtained from GTDB in Newick format was visualized with GraPhlAn v1.1.4^34^.

### Identification of l-asparaginase family members

Identification of l-asparaginase family members in Bacteria was simultaneously carried out at the protein sequence and protein domain levels. Sequence-based approach to identify l-asparaginases involved the BLAST reciprocal best hit method (RBH)^35^ to identify orthologous l-asparaginases. Specifically, the protein sequences of reference l-asparaginase types (i.e., EcAI, EcAII, EcAIII, ReAIV, and ReAV) were queried by BLAST v.2.9.0 + (e-value: 10^–3^)^36^ against proteomes of 45,555 bacterial species. The highest scoring protein obtained by BLAST in a given species was then used as a query in BLAST search against the proteome of bacterial species containing the reference protein (i.e., *E. coli* or *R. etli*). Pairs of l-asparaginase protein sequences that were reciprocally the best matches of each other in the two BLAST searches were considered orthologous.

The domain-based approach to identify l-asparaginases used Hidden Markov Models (HMM) of protein domains from Pfam database (v34.0, March 2021). HMMs were mapped using pfam_scan.pl^19^ as a wrapper for hmmscan (v3.3.1)^37^ with an *e*-value threshold of 10^–3^ (default in Pfam). Protein sequences containing at least one l-asparaginase domain (i.e., PF00710, PF01112, or PF06089) were aligned using water from EMBOSS package v6.6.0^38^ to the sequences of the five reference l-asparaginases (EcAI, EcAII, EcAIII, ReAIV, and ReAV), and were classified into one of the five l-asparaginase types based on the highest alignment score. Finally, the results of the two approaches were merged to yield the full list of orthologous l-asparaginase proteins.

### Over- and under-representation of l-asparaginase proteins in taxonomic units

Enrichment analysis of each l-asparaginase type was performed across all bacterial taxonomic units higher than species (i.e., genus, family, order, class, phylum). The number of finds of each l-asparaginase type in a given taxonomic unit was calculated as the number of species containing the protein to all species in a given taxonomic unit. A binomial test was used to compare the number of finds of each l-asparaginase type in each taxonomic unit in reference to the background frequency of that l-asparaginase type in Bacteria. *P*-values for under- and over-representation were calculated using cumulative distribution function (cdf) and survival function (1 − cdf), respectively, as implemented in SciPy v1.7.3^39^.

### Phylogenetic analysis

Protein sequences of each l-asparaginase type were aligned using Clustal Omega v1.2.4^40^. Phylogenetic trees were constructed using the maximum likelihood method and 1000 bootstrap replicates with MEGA software v.11.0.10^41^.

### Structure predictions, superpositions, and sequence conservation

The structural model for ReAIV was predicted with AlphaFold2^26^ and Robetta^27^ algorithms. The quality of the predictions was assessed using the pLDDT (predicted local distance difference test) and PAE (predicted aligned error) metrics, as illustrated in Supplementary Fig. S5.

Due to the relatively low overall sequence similarity, superpositions of protein structures and/or models generated by Robetta^27^ and AlphaFold2^26^ were calculated using the Secondary Structure Matching (SSM) tool^42^ from the ccp4 package^43^. The level of sequence identity mapped on the l-asparaginase protein structures (EcAI, EcAII, EcAIII, ReAIV, and ReAV) was assessed based on global pairwise sequence alignments between the prototypic enzyme and all its orthologous sequences from other bacterial species. The percent of identity was calculated separately for each residue of the aligned prototypic sequence (i.e., EcAI, EcAII, EcAIII, ReAIV, and ReAV).

## Supplementary Information

Supplementary Tables.

Supplementary Information.

## Data Availability

The datasets analyzed in the present study are available in the Genome Taxonomy Database (GTDB) release 202 (https://data.gtdb.ecogenomic.org/releases/release202/202.0/). All data generated in this study are available as Supplementary Information.
