# Supplementary material for: Massive annotation of bacterial l-asparaginases reveals their puzzling distribution and frequent gene transfer events
Source: Sci Rep. 2022 Sep 22;12:15797. doi: 10.1038/s41598-022-19689-1 (PMC9500103; doi:10.1038/s41598-022-19689-1)
Supplement: Supplementary file 2 — Supplementary Information. [file 41598_2022_19689_MOESM2_ESM.docx]

**Massive annotation of bacterial L-asparaginases reveals their puzzling distribution and frequent gene transfer events**

Zielezinski A, Loch JI, Karlowski WM, Jaskolski M.

**
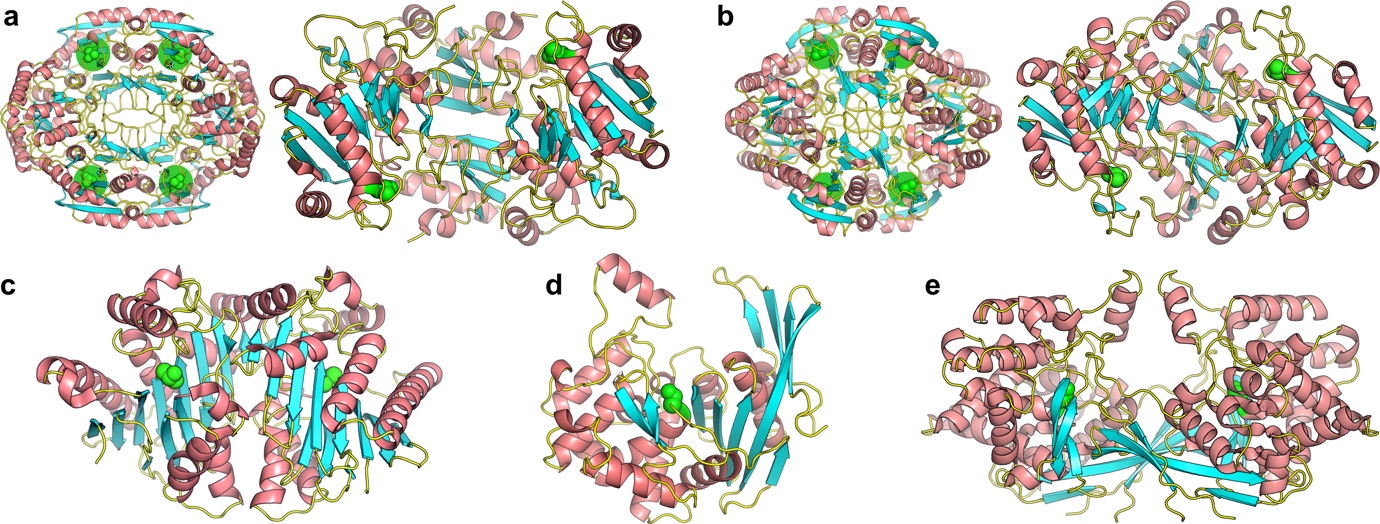
**

**Figure S1. Crystal structures of representative L-asparaginases. a.** EcAI and **b.** EcAII form homotetramers (dimers of the intimate dimers) presented on the left sides of panels (**a**) and (**b**); the intimate dimers are presented on the right of panels (**a**) and (**b**). **c.** Structure of the EcAIII homodimer (heterotetramer after autocleavage). **d.** Model of ReAIV obtained from the Robetta server. **e.** Structure of the ReAV homodimer. In all panels, the nucleophilic residue, Thr (in EcAI, EcAII and EcAIII) or Ser (in ReAIV and ReAV) is shown in light green space-filling representation. The presented structures are accessible in the PDB as: 2him (EcAI), 3eca (EcAII), 2zal (EcAIII), and 7os5 (ReAV).

**Figure S2. Protein domain content of five reference L-asparaginase types (EcAI, EcAII, EcAIII, ReAIV, and ReAV).** EcAI and EcAII contain two Pfam domains, namely Asparaginase N-terminal domain (PF00710) and Asparaginase C-terminal domain (PF17763). The sequences of EcAI and EcAII show 23.6% identity and 36.6% similarity. EcAIII contains Asparaginase_2 Pfam domain (PF01112). ReAIV and ReAV contain Asparaginase_II Pfam domain (PF06089) and share 30.7% / 44.1% sequence identity / similarity.

**Figure S3. Comparison of genome quality statistics between bacterial species with at least one type of L-asparaginase (ASNase^+^) and species without any L-asparaginase type (ASNase^-^).** Genome quality statistics for each genome were obtained from Genome Taxonomy Database (GtDB) release 202, including: **a.** Genome completeness (%) assessed by CheckM, **b.** L50 defined as a count of smallest number of contigs whose length sum makes up half of genome size, **c.** Number of different aa-tRNAs, **d.** Number of proteins, and **e.** MIMAG genome quality category. The differences of **a-d** distributions between ASNase^+^ and ASNase^-^ were tested using two-sided Mann–Whitney U-test, *P* < 10^-5^.

**Figure S4. Protein sequence similarity of five L-asparaginase types in bacteria.** For each L-asparaginase type (i.e., AI, AII, AIII, AIV, and AV), **a.** local and **b.** global pairwise alignments were computed between the reference asparaginase protein (e.g., EcAI) and its orthologous proteins. Box plots show the distribution of alignment scores, percentages of identity and similarity for AI (*n* = 13,130), AII (*n* = 12,698), AIII (*n* = 11,436), AIV (*n* = 8337), and AV (*n* = 1672). The horizontal line in each box marks the median; boxes indicate the first and third quartiles; whiskers mark the lowest and highest non-outlier values.


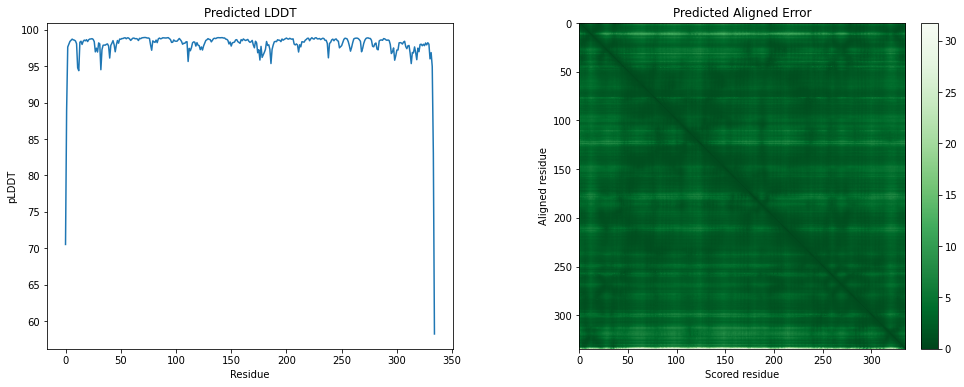


**Figure S5.** pLDDT and PAE metrics produced by AlphaFold-based ReAIV structure prediction.

**Amino acid sequences of reference EcAI, EcAII, EcAIII, ReAIV, and ReAV proteins**

>EcAI

MQKKSIYVAYTGGTIGMQRSEQGYIPVSGHLQRQLALMPEFHRPEMPDFTIHEYTPLMDS

SDMTPEDWQHIAEDIKAHYDDYDGFVILHGTDTMAYTASALSFMLENLGKPVIVTGSQIP

LAELRSDGQINLLNALYVAANYPINEVTLFFNNRLYRGNRTTKAHADGFDAFASPNLPPL

LEAGIHIRRLNTPPAPHGEGALIVHPITPQPIGVVTIYPGISADVVRNFLRQPVKALILR

SYGVGNAPQNKAFLQELQEASDRGIVVVNLTQCMSGKVNMGGYATGNALAHAGVIGGADM

TVEATLTKLHYLLSQELDTETIRKAMSQNLRGELTPDD

>EcAII

MEFFKKTALAALVMGFSGAALALPNITILATGGTIAGGGDSATKSNYTAGKVGVENLVNA

VPQLKDIANVKGEQVVNIGSQDMNDDVWLTLAKKINTDCDKTDGFVITHGTDTMEETAYF

LDLTVKCDKPVVMVGAMRPSTSMSADGPFNLYNAVVTAADKASANRGVLVVMNDTVLDGR

DVTKTNTTDVATFKSVNYGPLGYIHNGKIDYQRTPARKHTSDTPFDVSKLNELPKVGIVY

NYANASDLPAKALVDAGYDGIVSAGVGNGNLYKTVFDTLATAAKNGTAVVRSSRVPTGAT

TQDAEVDDAKYGFVASGMLNPQKARVLLQLALTQTKDPQQIQQIFNQY

>EcAIII

MGKAVIAIHGGAGAISRAQMSLQQELRYIEALSAIVETGQKMLVAGESALDVVTEAVRLL

EECPLFNAGIGAVFTRDETHELDACVMDGNTLKAGAVAGVSHLRNPVLAARLVMEQSPHV

MMIGEGAENFAFAHGMECVSPEIFSTPLRYEQLLAAREEGATVLDHSGAPLDEKQKMGTV

GAVALDLDGNLAAATSTGGMTNKLPGRVGDSPLVGAGCYANNASVAVSCTGTGEVFIRAL

AAYDIAALMDYGGLSLAEACERVVMEKLPALGGSGGLIAIDHEGNVALPFNTEGMYRAWG

YAGDTPTTGIYREKGDTVATQ

>ReAIV

MTNPVTVEVTRGLLVESRHRGAVAVVDGDGKLFFSLGDIDTAVFPRSACKAMQALPLVES

GAADAYGFGDKELALACASHNGEEEHVALAASMLSRAGRNVEALECGAHWSMNQKVLIQQ

ARSLDAPTALHNNCSGKHAGFICACCHRDIDPKGYVGYEHPLQVEIRAVMERLTGAVLGA

ESCGTDGCSIPTYAMPLRNLAHGFARMATGTGLEPLRAKASRRLIEACMAEPFYVAGSGR

ACTKLMQIAPGRIFVKTGAEGVFCAAIPEKGIGISLKSEDGATRAAEAMVAATLARFFET

EETVHAALMAFAAMPMRNWNGIHVGDIRATSVFSA

>ReAV

MEREMTPSEDFVVTDRGGIVENSHRVHAAVVDAKGRLLYALGNPTRMTLARSAAKPAQAL

AILETEGVAGYGFDDADIALMCASHSSEDRHIARTRAMLSKIKAEEADLRCGGHPSLSEM

VNRSWIKQDFIPTAVCSNCSGKHVGMLAGARAIGAGTDGYHLPDHPMQGRVKRTVAELCD

LDAGDVEWGTDGCNLPTPAFPLDRLGRIYAKLASAADGSDAGEGQSTRCAALAHIFRAMA

RHPEMVAGEGRYCTMLMRAFDGALVGKLGADASYAIGVRASDATRQLGTDGALGISVKIE

DGNLEMLYAVVTELLERLGIGSPDVRSQLASFHHPQRVNTMGVTTGGVSFPFKLRGSKSN

VDDPRLAAVAR
